# Hitting pause on the cell cycle

**DOI:** 10.7554/eLife.46781

**Published:** 2019-04-09

**Authors:** Thomas Eekhout, Lieven De Veylder

**Affiliations:** 1 Department of Plant Biotechnology and BioinformaticsGhent UniversityGhentBelgium; 2 VIB Center for Plant Systems BiologyGhentBelgium

**Keywords:** cell cycle arrest, DNA damage, heat stress, transcription factors, protein stability, gene expression, *A. thaliana*

## Abstract

Two recently discovered transcription factors stop cells from dividing when plants face extreme heat and DNA damage.

**Related research article** Takahashi N, Ogita N, Takahashi T, Taniguchi S, Tanaka M, Seki M, Umeda M. 2019. A regulatory module controlling stress-induced cell cycle arrest in *Arabidopsis*. *eLife* **8**:e43944. doi: 10.7554/eLife.43944

When something goes awry during the cell cycle – for example, if DNA gets broken during replication – checkpoint mechanisms put the cycle on pause so that the cell can repair the damage before dividing. In mammals, failure to activate these checkpoints can lead to cancer.

The p53 tumor suppressor is a mammalian transcription factor which controls the genes that stop the cell cycle, repair DNA, and even trigger cell death in response to DNA damage (Kastenhuber and Lowe, 2017). Many cell cycle and DNA repair genes are conserved between vertebrates and plants, yet a p53 ortholog has never been found in any plant genome sequence. Instead, plants use SOG1 (short for suppressor of gamma-response 1), a plant-specific transcription factor that also arrests the cell cycle, coordinates DNA repair and promotes cell death.

Recently, two independent studies have demonstrated that SOG1 regulates the expression of almost all the genes that are induced when DNA is damaged, including other transcription factors from the same family (Bourbousse et al., 2018; Ogita et al., 2018). Now, in eLife, Masaaki Umeda and colleagues from the Nara Institute of Science and Technology, the RIKEN Center for Sustainable Resource Science and the RIKEN Cluster for Pioneering Research – with Naoki Takahashi as first author – report on the roles of two of these SOG1-like transcription factors, ANAC044 and ANAC085 (Takahashi et al., 2019).

In plants, SOG1 can bind to the promoter regions of these factors, and it encourages the transcription of these genes upon DNA damage. Knockout experiments show that the ANAC044 and ANAC085 proteins are not necessary to repair DNA; instead, they stop the cell cycle just before division by increasing the levels of transcription factors called Rep-MYBs (where Rep is short for repressive). Once stabilized, these factors can bind to and inhibit genes involved in the progression of cell division (Ito et al., 2001). When the cells are ready to divide, Rep-MYBs are marked for destruction, freeing up the genes that promote division so that they can be activated by other transcription factors (Chen et al., 2017).

Rep-MYBs do not accumulate when the genes for ANAC044 and ANAC085 are knocked out. The roots of mutant plants that lack both of these genes can therefore keep growing when agents that damage DNA are present. However, these double knockouts do not show a difference in the levels of RNA transcripts of Rep-MYBs. This prompted Takahashi et al. to speculate that an intermediate molecular step allows ANAC044 and ANAC085 to control the levels of Rep-MYBs after transcription, possibly by inhibiting the machinery that labels and degrades these proteins.

Upon DNA damage, two kinases called ATM and ATR phosphorylate specific sites on SOG1 so that it can bind to DNA and perform its regulatory role (Sjogren et al., 2015; Yoshiyama et al., 2013; Ogita et al., 2018). Both ANAC044 and ANAC085 have sequences that are very similar to those of SOG1, but they appear to lack these phosphorylation sites. Moreover, overexpression of ANAC044 only inhibits the cell cycle if the DNA is damaged. It is therefore possible that this transcription factor only works in the presence of ANAC085, or that its activity is controlled by other kinases.

Overall, the work by Takahashi et al. shows that plants have harnessed SOG1-like transcription factors to regulate the network of genes that respond to DNA damage. These results represent a major step in unraveling the hierarchical control of the DNA damage response in plants. So far, SOG1 appears to be the master regulator, delegating downstream responses among various regulators (Figure 1), with ANAC044 and ANAC085 stopping the cell cycle before division. Takahashi et al. also report that when plants are exposed to high temperatures, ANAC044 and ANAC085 help to halt the cell cycle. Therefore, these two transcription factors could be part of a central hub that delays cell division in response to a diverse set of stresses.

**Figure 1.**
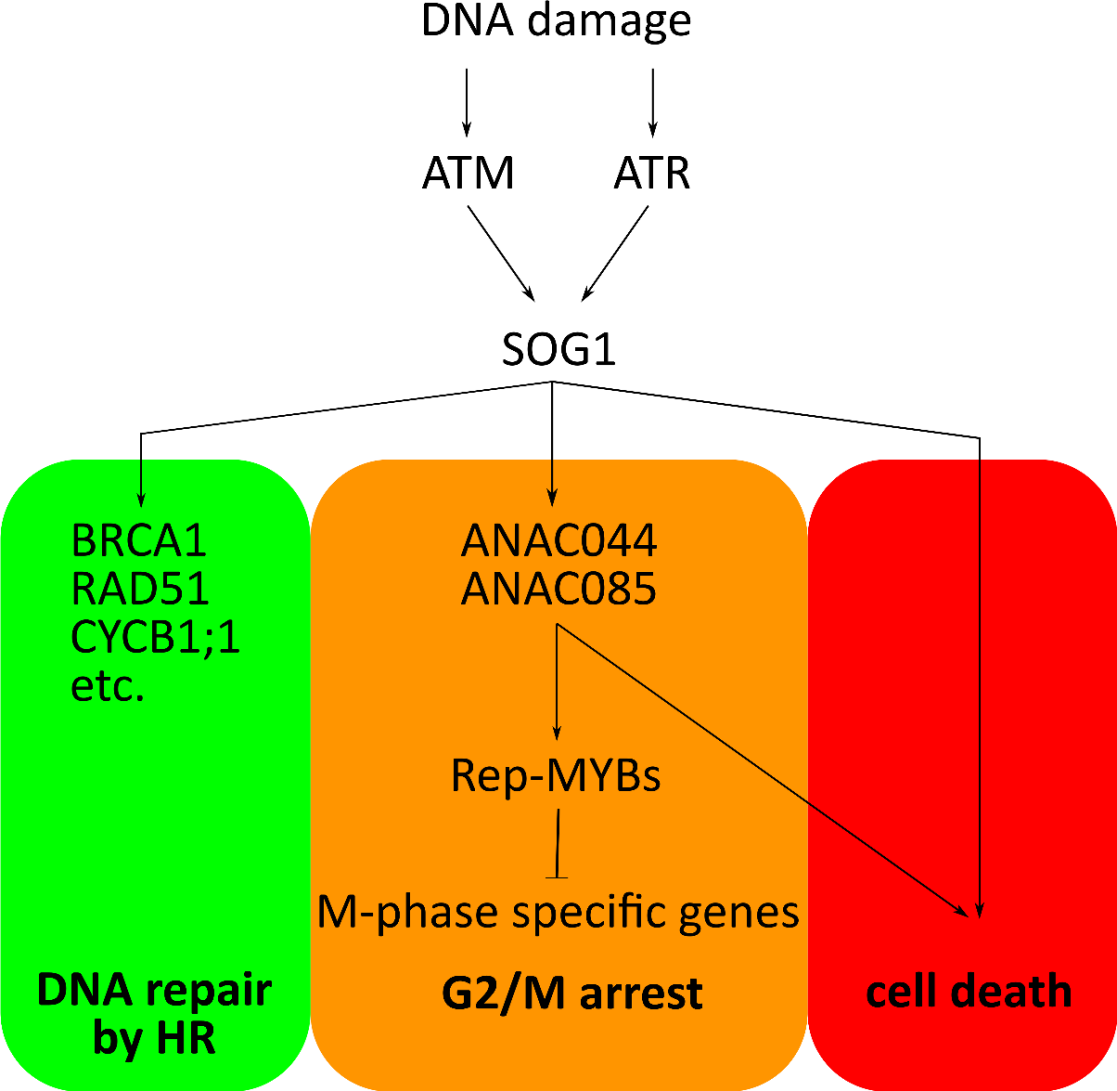
Hierarchical control of the DNA damage response in plants. In plant cells, the kinases ATM and ATR are activated by different types of DNA damage. These enzymes go on to phosphorylate and activate the SOG1 transcription factor, which then binds to and switches on its target genes. These include (**i**) genes involved in DNA repair through homologous recombination (HR); (**ii**) the genes for ANAC044 and ANAC085, the newly identified transcription factors that help to stop the cell cycle; (**iii**) genes that trigger a cell death program (for when damage is too severe). ANAC044 and ANAC085 work by increasing the levels of Rep-MYB transcription factors. If stabilized, these proteins maintain the cells in the phase just before division (G2/M arrest) by binding to and repressing the genes essential for cell division to proceed. It is still unclear how Rep-MYBs are stabilized, or how SOG1 and ANAC044/ANAC085 may trigger cell death (Takahashi et al., 2019).
